# Evaluation of Phytocannabinoids as Antitumor Agents against Ovarian Cancer Cells in Combination with Carboplatin

**DOI:** 10.1021/acsomega.6c04745

**Published:** 2026-08-26

**Authors:** Uri Warter, Yuval Yogev, David C. Nwobodo, Ksenia Lialin, Danielle Mantin, Mihai Meirovitz, Michael M. Meijler, Amit Mayer

**Affiliations:** † The Joyce & Irving Goldman Medical School, Faculty of Health Sciences, 85456 Ben-Gurion University of the Negev, Be’er Sheva 8410501, Israel; ‡ The Morris Kahn Laboratory of Human Genetics at the National Institute of Biotechnology in the Negev and Faculty of Health Sciences, Ben-Gurion University, Be’er Sheva 8410501, Israel; § Department of Chemistry and the National Institute for Biotechnology in the Negev, 26732 Ben-Gurion University of the Negev, Be’er Sheva 8410501, Israel; ∥ Division of Obstetrics and Gynecology, Gynecology Oncology Unit, Faculty of Health Sciences, Soroka University Medical Center, Ben-Gurion University of the Negev, Be’er Sheva 8410501, Israel

## Abstract

Ovarian cancer is the second leading cause of death from gynecologic malignancy worldwide. The integration of phytocannabinoids into oncological care has outpaced our mechanistic understanding of their interactions with frontline chemotherapeutics. In this study, we provide a detailed pharmacological evaluation of two phytocannabinoids, Δ9-tetrahydrocannabinol (THC) and cannabidiol (CBD), as antineoplastic agents against three epithelial ovarian cancer (EOC) cell lines. Using established EOC cell models, we demonstrate that both cannabinoids exhibit intrinsic antiproliferative activity, but CBD monotherapy significantly outperforms CBD-THC combinations. Furthermore, RT-qPCR and in silico analyses revealed a low density of classical receptors, indicating that cannabinoid efficacy in EOC may be primarily driven by receptor-independent mechanisms. Most importantly, we report that the coadministration of cannabinoids with carboplatin, the standard of care for EOC, paradoxically inhibits the platinum agent’s antineoplastic efficacy. Our results challenge the “entourage effect” in an oncological context and provide a necessary cautionary framework for the use of cannabinoids alongside specific platinum-based regimens. Given the increasing use of cannabis among cancer patients, further robust studies examining interactions between cannabis and conventional chemotherapeutic agents are required to better evaluate safety and therapeutic efficacy in EOC treatment.

## Graphical abstract

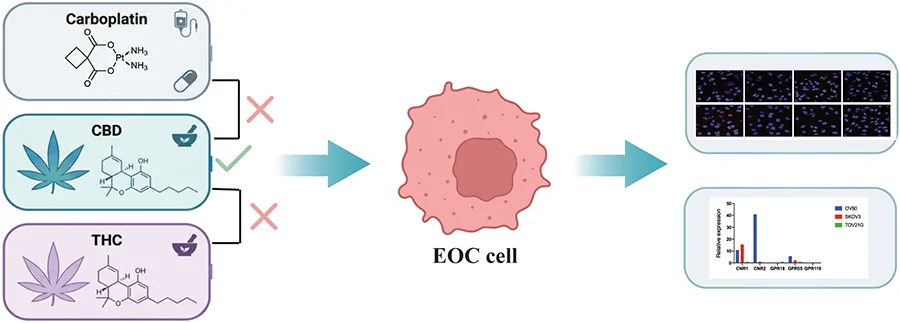

## 1 Introduction

Ovarian cancer remains the most lethal gynecological malignancy, primarily due to the high frequency of late-stage diagnosis and the inevitable development of resistance to standard platinum-based therapies.^1^ By 2050, this disease is projected to rise globally to about 425,086 new cases and an estimated 269,842 deaths annually.^2,3^ Ovarian cancer is a heterogeneous disease encompassing many different subtypes, yet close to 90% of cases are of epithelial origin.^4^ Epithelial ovarian cancer (EOC) is highly chemo-sensitive, and approximately 70% of ovarian carcinoma patients are expected to respond to platinum-based chemotherapy as the first line of treatment.^5^ Interestingly, several studies have identified the endocannabinoid system (ECS) as a potential metabolic vulnerability in ovarian cancer.

The ECS is a critical regulatory network within the central nervous system, and its signaling pathway is mainly mediated by the activation of the G-protein-coupled receptors (GPCRs) CB1 and CB2. CB1 receptors are primarily distributed within the central nervous system, whereas the more peripheral CB2 receptors are found mainly in the immune system and the gastrointestinal tract.^6,7^ In addition, several other GPCRs, such as GPR55, GPR119, and GPR18, have been demonstrated to be activated by cannabinoid ligands.^8,9^ Ligands that activate the endocannabinoid system (ECS) can be either endogenous or exogenous. Endogenous ligands are termed endocannabinoids, whereas exogenous ligands are collectively referred to as cannabinoids. These include phytocannabinoids, over 100 compounds isolated from *Cannabis sativa*, as well as synthetic agents that interact with ECS receptors.^10^ Among the numerous phytocannabinoids identified to date, two have attracted particular attention over the past decades: Δ9-tetrahydrocannabinol (THC), the primary psychoactive constituent of *Cannabis sativa*, and cannabidiol (CBD), a nonpsychoactive phytocannabinoid. Both compounds have been extensively studied for their therapeutic potential, with CBD receiving particular interest due to its lack of psychoactive effects.^11,12^ A recent study by Tong et al.^13^ has highlighted the ability of CBD and THC to disrupt the PI3K/AKT/mTOR signaling axis, a pathway frequently exploited by ovarian cancer cells to drive proliferation and chemoresistance.

While the clinical use of phytocannabinoids, specifically CBD and THC, has surged among oncology patients for palliative care, their direct pharmacodynamic role in tumor suppression and their compatibility with frontline chemotherapeutics remain poorly defined.^14^ Concurrently, an increasing cohort of oncological patients utilizes cannabis as complementary alternative medicine (CAM) for the management of refractory symptoms, including chronic pain, cachexia, and anxiety. Many of these therapies have not yet been evaluated in clinical trials, and little is known about their interactions with conventional drugs. One of the earliest investigations pointing to the antineoplastic effect of cannabinoids was conducted in 1975, when Munson and colleagues demonstrated that cannabinoids inhibited lung adenocarcinoma proliferation in vitro and in murine models in vivo.^15^ Since then, many mechanisms have been proposed for the antitumorigenic effect of cannabinoids, and their cytotoxicity has been reported in various cancer types.^16–18^ In addition, several studies have explored combining conventional chemotherapeutic drugs with cannabinoids to achieve a synergistic effect.^19–21^ Chen et al.^22^ recently reported that certain cannabinoids and their analogs may act as cisplatin sensitizers, potentially lowering the required therapeutic dose of these highly toxic agents.

Despite these promising reports, two key questions remain unresolved in the medicinal chemistry of cannabinoids. First, it is unclear whether their anticancer activity depends on activation of the classical G-protein-coupled receptors CB1 and CB2, as accumulating evidence suggests the involvement of receptor-independent pathways such as ferroptosis induction and modulation of intracellular oxidative stress. Second, while synergy has been reported with cisplatin, the interactions between cannabinoids and carboplatin, the most commonly prescribed platinum agent for EOC, have not been rigorously characterized.

In light of emerging new data revealing the role of the endocannabinoid system in female reproductive tissues,^23,24^ we provide a comprehensive pharmacological evaluation of CBD and THC in three ovarian cancer cell line models. We investigate the comparative efficacy of mono vs combined cannabinoid treatments, analyze receptor expression profiles to determine therapeutic relevance, and, critically, evaluate the compatibility of these compounds with carboplatin.

## 2 Results and Discussion

### 2.1 Potential of CBD and THC as Antineoplastic Agents against EOC

In light of the growing data on the cytotoxic effects of cannabinoids across various cancer types and the role of the endocannabinoid system in female reproductive tissue,^23–25^ this study was initiated to explore the cytotoxic properties of two phytocannabinoids in a model using three EOC cell lines. Prior to experimental evaluation, the baseline carboplatin sensitivity profile across the three EOC cell lines was established. TOV-21G, OV-90, and SKOV-3 cells were grown and treated with carboplatin at concentrations ranging from 0.05 to 160 μM. Cell metabolic activity and viability were quantified utilizing the XTT assay, and dose response curves were plotted (Figure 1). In line with previously reported observations, the TOV-21G cell exhibited the highest sensitivity to carboplatin with an IC_50_ of 10.5 μM. The OV-90 cell line was moderately sensitive to carboplatin with an IC_50_ of 18.3 μM, and the SKOV-3 cell line was the least sensitive to carboplatin with an IC_50_ of 35.5 μM (Table S2).

**Figure 1:**
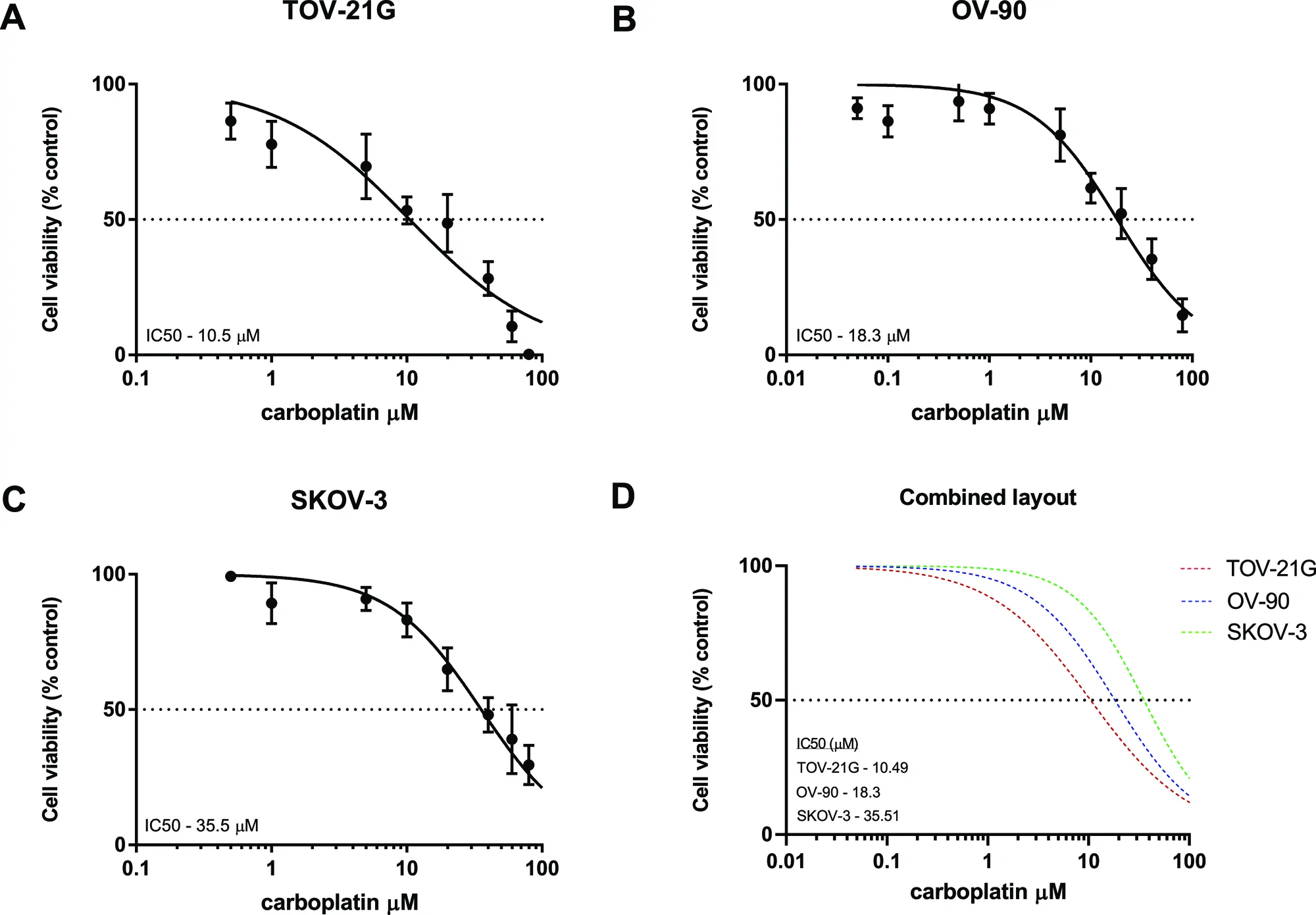
Dose response curves of carboplatin on EOC cells. A, B, and C represent the IC_50_ values of carboplatin on TOV-21G, OV-90, and SKOV-3 cell lines, respectively. (D) represents an overlay comparison analysis of the effect of carboplatin on the three cell lines. Each data point represents the mean of at least three separate experiments, and IC_50_ values were significantly different between all 3 cell lines (PV < 0.0001).

To assess whether CBD, THC, and their mixture possess an antineoplastic effect on EOC cells, the three representative EOC cell lines were cultured and exposed to the respective cannabinoids at concentrations ranging from 0.05 to 100 μM. Cellular activity was then examined using the XTT viability assay, and dose–response curves were plotted (Figure S1). Both CBD and THC, as monotherapies and in combination, demonstrated significant antineoplastic efficacy, consistently suppressing the viability of ovarian cancer cells. Of the three, CBD showed the strongest cytotoxicity against the carboplatin-sensitive and intermediate cell lines we investigated. The TOV-21G and OV-90 cell lines were the most inhibited by CBD, with IC_50_ values of 7.1 μM and 18.6 μM, respectively. In contrast, the SKOV-3 cell line exhibited resistance to both CBD and THC (Table 1). We found that EOC cell lines’ sensitivity to carboplatin correlated with their sensitivity to CBD and THC, as evidenced by differences in IC_50_ values. The TOV-21G was the most sensitive cell line to both carboplatin and cannabinoids, and the SKOV-3 was the least sensitive (Figure 2).

**Figure 2:**
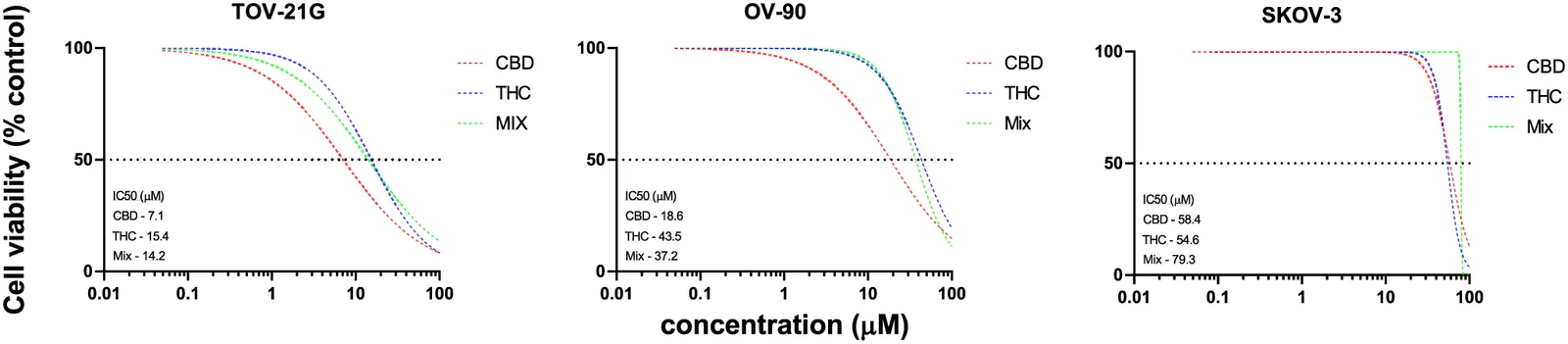
Overlay of the cytotoxicity of each cannabinoid and their mixture on TOV-21G, OV-90, and SKOV-3 cell lines. Each data point represents the mean of at least three separate experiments, and IC_50_ values were significantly different between all 3 cell lines (PV < 0.0001).

**Table 1:** IC_50_ Values of CBD, THC, and Their Mixture (1:1) on EOC Cell Lines^a^

IC_50_ (μM)	TOV-21G	OV-90	SKOV-3
CBD	7.09 ± 1.02	18.61 ± 3.55	58.44 ± 4.04
THC	15.38 ± 1.55	43.50 ± 4.53	54.63 ± 4.56
Mix	14.15 ± 2.87	37.17 ± 6.25	79.32 ± 2.59

a Values represent the mean ± SD of at least 3 different experiments.

These data are of clinical relevance given the therapeutic potential of CBD as an antineoplastic agent devoid of adverse psychotropic effects.^26^ Furthermore, the effects of CBD and THC were similar on the carboplatin-resistant cells. We also found that EOC cells’ resistance to carboplatin corresponded with the resistance to CBD and THC. The therapeutic potential of cannabinoids in epithelial ovarian cancer (EOC) lies in their ability to selectively target malignant cell signaling pathways. In line with our findings, recent studies by Tong et al.^13^ and Chen et al.^22^ demonstrate that CBD and THC significantly suppress the viability of A2780 and SKOV3 cell lines. The antineoplastic mechanism involves inhibiting the PI3K/AKT/mTOR pathway, a central driver of ovarian cancer progression, and restoring PTEN expression, which is often lost in aggressive EOC phenotypes.^13^ Furthermore, CBD has been shown to induce ferroptosis, an iron-dependent form of cell death, providing a multifaceted approach to eliminating cancer cells.^22^

### 2.2 Comparative Cytotoxicity of THC and CBD in Combination Therapy

Inspired by reports suggesting that combinations of cannabinoids exert greater cytotoxic effects on cancer cells than individual cannabinoids alone,^20,27–30^ we investigated the effect of treating EOC cells with a combination of CBD and THC compared with treatment with each phytocannabinoid individually. While literature often explores the “entourage effect,” our findings indicate that CBD exhibits superior cytotoxicity as a monotherapy compared to its combination with THC. This observation suggests a potential antagonistic interaction between the two cannabinoids in specific EOC models. Our results did not show that the combination was more cytotoxic (Figure 2). THC appears to be less active against EOC cells and does not exhibit synergistic effects when combined with CBD. Analyzing the results reveals that when the TOV-21G and OV-90 cell lines were treated with a 1:1 mixture of CBD and THC, the IC_50_ was mainly determined by the CBD. The IC_50_ value of the CBD and THC mixture on TOV-21G cells was 14.2 μM, exactly twice that of CBD alone. This indicates that, when combining the two cannabinoids, the cytotoxic effect was largely dependent on CBD concentration, with minimal synergistic or antagonistic effects from THC (Table 1). Although Tong et al.^13^ reported synergy at an equimolar (1:1) ratio, they also noted that the interaction is highly ratio-dependent. If the concentration of THC interferes with CBD’s ability to downregulate oncogenic signaling or if they compete for noncanonical binding sites, the resulting antiproliferative effect may be diminished. This underscores the importance of phytocannabinoid purity and precise formulation of ratios in medicinal chemistry.

### 2.3 Expression Analysis of Cannabinoid Receptors

The clinical utility of cannabinoid-based therapies is traditionally evaluated by measuring the expression of classical G-protein-coupled receptors (GPCRs), specifically CB1 (CNR1) and CB2 (CNR2). Our findings reveal that CNR1 (which encodes the CB1 receptor) is predominantly abundant in normal ovarian tissue (Figure 3), while CNR2 exhibits consistently low expression across all epithelial ovarian cancer cell lines. However, the most abundant receptor in cancer cells was GPR55, which is expressed at low levels in normal ovarian tissue. These expression differences between cell lines were demonstrated by RT-qPCR analysis (Figure 3A–B). While these transcriptomic data suggest a divergence from classical signaling, these observations remain correlative, and further functional knockdown assays are required to definitively establish a causal link. Still, our data suggest that the classical endocannabinoid system may not be the primary mediator of cannabinoid-induced cytotoxicity in malignant ovarian cells. Consistent with this notion, studies by Chen et al.^22^ and Tong et al.^13^ report that the antiproliferative effects of CBD and THC frequently occur independently of classical cannabinoid receptor signaling. While Tong et al.^13^ attributed these effects to inhibition of the PI3K/AKT/mTOR pathway, our findings instead point to the orphan receptor GPR55 as a potential determinant of the therapeutic window. Notably, GPR55 was the most abundantly expressed receptor in EOC cells, whereas its expression remained low in normal ovarian tissue (Figure 3A).

**Figure 3:**
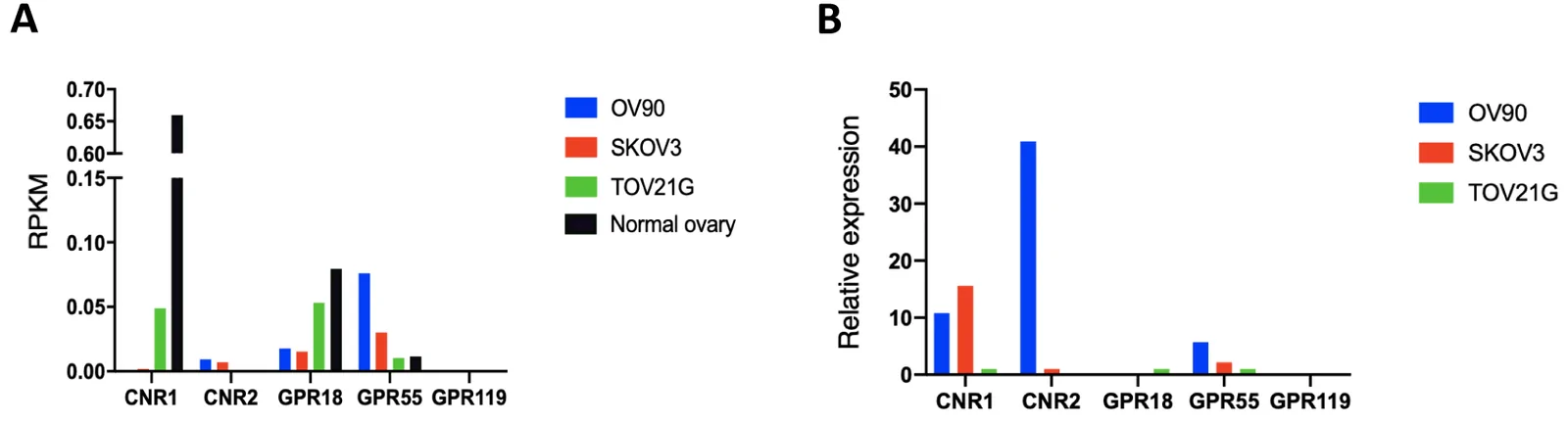
Expression of cannabinoid receptors in normal ovarian tissue and EOC cell lines. (A) RNA-seq data gathered from GTEx (*n* = 180) and CCLE. (B) Expression as analyzed by RT-qPCR of untreated cells, relative to GAPDH expression. Results are normalized to the cell line with the lowest expression of that gene. Experiments were carried out in triplicate (*n* = 3).

The significance of GPR55 expression is underlined by its role in oncogenic signaling. Previous studies by Piñeiro et al.^31^ demonstrated that GPR55 is a pro-proliferative driver in ovarian cancer and that its activity is directly inhibited by CBD. This provides a plausible, though tentative, mechanistic framework for our sensitivity assays, where CBD monotherapy exhibited superior cytotoxicity compared to THC or the CBD-THC (1:1) mixture. Because THC acts as an agonist for classical cannabinoid receptors (which are low in these cells) but lacks the potent GPR55-antagonistic profile of CBD, its efficacy is diminished.^22,32^ The differential transcription of GPR55 in immortalized EOC models relative to normal tissue (Figure 3) suggests a potential therapeutic window; however, this differential expression must be validated in primary patient-derived tissues to confirm clinical utility. This “receptor-decoupled” efficacy model suggests that future medicinal chemistry efforts should not only focus on classical CB1/CB2 modulation, but also on the CBD-GPR55 axis to selectively inhibit ovarian cancer cell proliferation while sparing normal ovarian tissue.

### 2.4 Interaction between Cannabinoids and Carboplatin Efficacy

Given the modest cytotoxicity against EOC cells shown above and the increasing use of cannabinoids in therapeutic settings, particularly among cancer patients, we decided to examine whether a combination of carboplatin and cannabinoids could exhibit synergistic effects. We first aimed to determine whether cannabinoids have the ability to augment carboplatin activity against EOC cells by comparing the IC_50_ value of carboplatin in the absence and in the presence of CBD, THC, and their mixtures. A pivotal finding in our study is that the combination of cannabinoids with carboplatin appears to inhibit carboplatin’s antineoplastic activity. Across all three cell lines, suboptimal concentrations of cannabinoids did not sensitize cells to carboplatin. In fact, IC_50_ values of all trials in the presence of cannabinoids were higher than those with carboplatin alone (Figure 4A–C). The negative effect on carboplatin cytotoxicity in the TOV-21G and OV-90 cell lines was minimal, with only one trial demonstrating a significant effect in each cell line (PV < 0.05). The carboplatin-resistant SKOV-3 cell line was most negatively affected by the cannabinoids and exhibited higher IC_50_ values in all trials with significant statistical differences when combining carboplatin with CBD and THC individually (Figure 4C).

**Figure 4:**
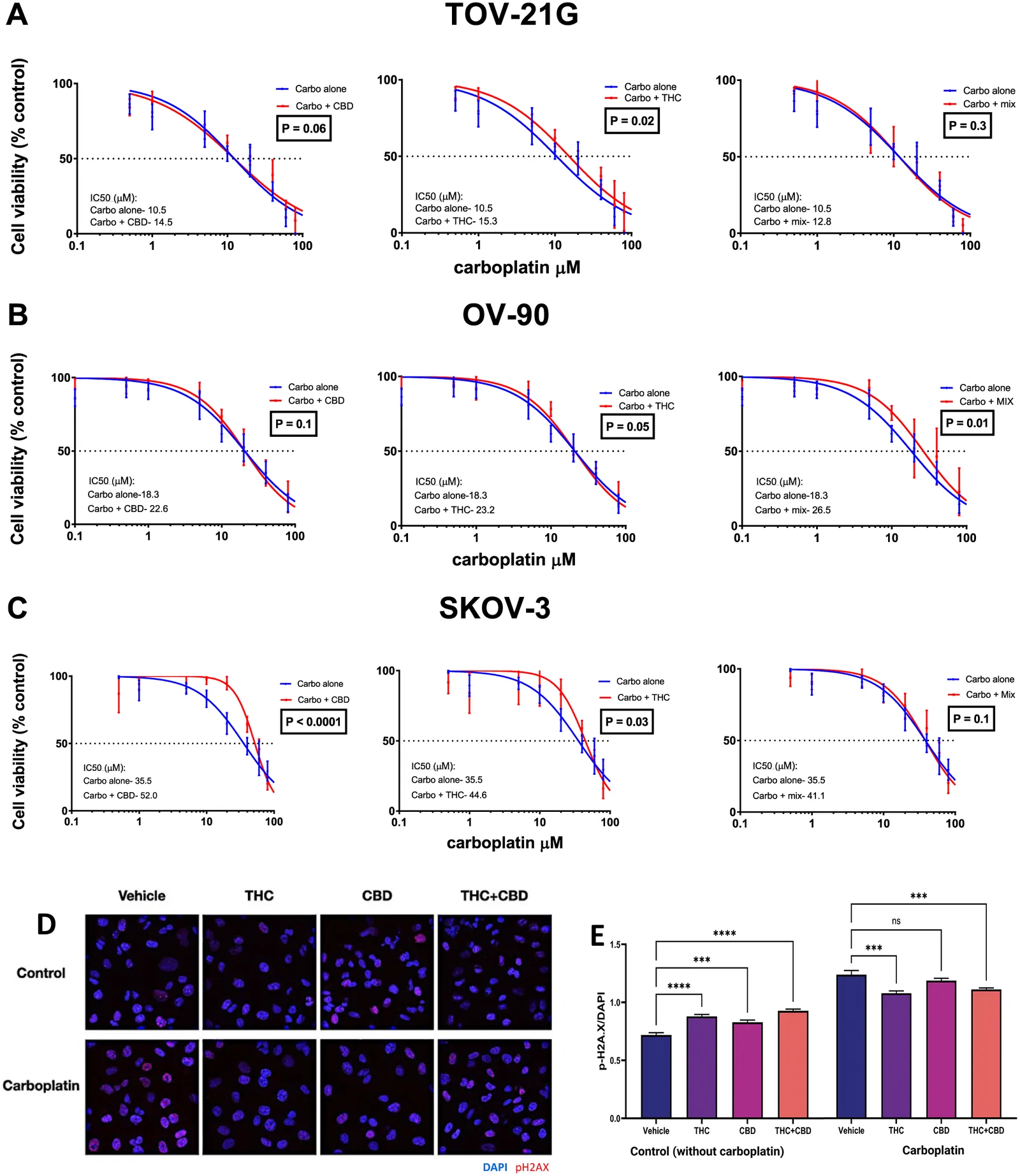
Assessment of the modulatory effect of cannabinoids on the efficacy of carboplatin. Comparison of the IC_50_ value of carboplatin in the presence of each suboptimal cannabinoid vs carboplatin alone for TOV-21G (A), OV-90 (B), and SKOV-3 (C) cell lines. (D) represents the immunofluorescence pattern of TOV-21G cells after treatment with suboptimal levels of cannabinoids or vehicle (ethanol) with or without carboplatin at its IC_50_ concentration. (E) represents the fluorescence intensity ratio of pH2A.X, representing DNA damage, normalized to DAPI, representing total DNA. Results were generated from scans of 3 ibidi 8-well μ-slides.

To further assess the modulatory effect of THC and CBD on carboplatin efficacy, we examined the DNA damage produced by carboplatin on TOV-21G cells in the presence and in the absence of suboptimal cannabinoid concentrations. Histone H2A.X becomes phosphorylated in response to double-stranded DNA breaks and consequently has been shown to undergo phosphorylation in cells exposed to carboplatin.^33^ If cannabinoids interfere with the activities of carboplatin, we should observe less phosphorylation of histone H2A. Cells were treated with carboplatin and cannabinoids, then stained with antibodies that specifically label phosphorylated H2A, and evaluated by immunofluorescence confocal microscopy (Figure 4D). TOV-21G cells treated with carboplatin alone clearly exhibit DNA damage, whereas in the presence of a suboptimal concentration of cannabinoids, they exhibit a significant weakening of H2A.X staining (Figure 4E).

To evaluate the pharmacological interactions between the cannabinoids and carboplatin, combination assays were performed at fixed potency ratios to characterize additive, synergistic, or antagonistic behavior. EOC cells were treated with cannabinoids and carboplatin at fixed ratios of their respective IC_50_ values, and a CI value was calculated for each combination using the median-effect analysis. A combination index (CI) was calculated to assess the nature of the interaction, where CI = 1 indicates an additive effect, CI < 1 a synergistic effect, and CI > 1 an antagonistic effect.^34^ Quantitative analysis revealed that all evaluated combinations yielded a CI > 1, indicating a uniform antagonistic interaction between the cannabinoids and carboplatin (Figure 5 A–D). A similar outcome was reported by Chen et al.^22^ for natural CBD and a different platinum compound, cisplatin. However, they identified synergy between syntheticCBD derivatives and cisplatin. This discrepancy may be attributed either to the different pharmacokinetic and pharmacodynamic profiles of carboplatin versus cisplatin or to interactions between the synthetic CBD derivatives and cisplatin. Mechanistically, if cannabinoids exert a cytostatic effect that induces cell cycle arrest, they may paradoxically desensitize malignant cells to carboplatin, which requires active cell division to induce DNA cross-linking and apoptosis. Such an inhibitory interaction highlights a significant clinical risk, suggesting that cannabinoid supplementation could potentially interfere with standard-of-care platinum therapies in EOC.

**Figure 5:**
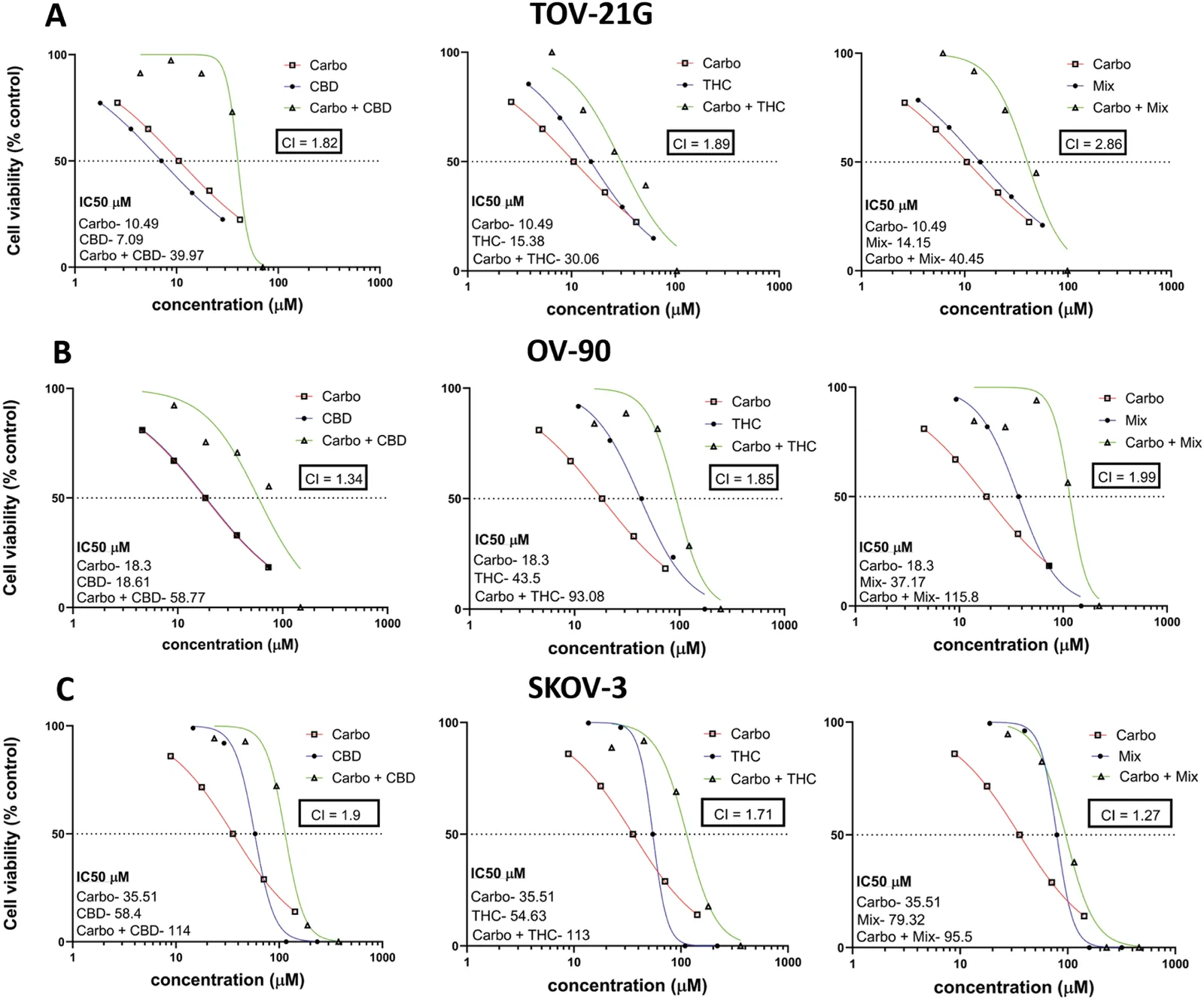
Median effect analysis of cannabinoid-carboplatin combination using a 16-data-point test. Figures represent the combination index (CI) values for TOV-21G (A), OV-90 (B), and SKOV-3 (C) cell lines. (D) represents the median effect analysis plots of the cell lines. Synergistic interactions are characterized by points under the dotted line, whereas antagonistic interactions are characterized by points above the dotted line. Each data point represents the mean of at least three separate experiments.

It is important to highlight that one limitation of this study is the use of in vitro cell lines alone. Also, the use of pure extracts of CBD and THC does not represent the overall variety of molecules that reach the blood when patients use cannabis or its extracts. Additionally, the concentrations used do not necessarily represent the physiological concentrations that reach the blood in the various ways that cannabis can be consumed.

## 3 Conclusion

In summary, while CBD and THC demonstrate individual promise as antineoplastic agents, their clinical application remains complex. While our studies show clear inhibitory effects of cannabinoids on ovarian cancer cells, their coadministration with carboplatin results in antagonistic interactions that compromise platinum-mediated cytotoxicity, and further investigations are needed to examine this interaction and elucidate possible mechanisms of action. While our studies show clear inhibitory effects of cannabinoids on ovarian cancer cells, our findings suggest that CBD monotherapy exhibits superior therapeutic efficacy compared to multicannabinoid combinations, and, crucially, that cannabinoids may antagonize the efficacy of carboplatin. Further investigations are needed to examine this interaction and elucidate possible mechanisms of action. Future work should encompass expanded evaluations across diverse EOC cell lines, ex vivo patient-derived tumor biopsies, and translational in vivo models. In addition, medicinal chemistry efforts should focus on structural optimization of CBD to enhance receptor-independent pathways, while carefully screening for potential adverse drug–drug interactions with standard chemotherapeutics.

## 4 Materials and Methods

### 4.1 Cell Culture and Reagents

We investigated 3 different human ovarian cancer cell lines according to their sensitivity to carboplatin as described previously;^35^ a carboplatin sensitive TOV-21G cell line (ovarian adenocarcinoma), an intermediately sensitive to carboplatin OV-90 cell line (ovarian papillary serous adenocarcinoma) and a carboplatin resistant SKOV-3 cell line (ovarian adenocarcinoma). All cell lines were purchased from the American Type Culture Collection (ATCC). TOV-21G and OV-90 cell lines were grown in a 1:1 mixture of MCDB 105 medium containing a final concentration of 1.5 g/L sodium bicarbonate, and medium 199 containing a final concentration of 2.2 g/L sodium bicarbonate. The medium was supplemented with fetal bovine serum to a final concentration of 15%, 2 mM l-glutamine and 1% penicillin-streptomycin solution. The SKOV-3 cell line was grown in McCoy’s 5a Medium Modified supplemented with fetal bovine serum to a final concentration of 10%, 2 mM l-glutamine and 1% penicillin-streptomycin solution. All cell lines were incubated in a humidified atmosphere with 5% CO2 at 37 °C and discarded after no more than 15 passages. Carboplatin (Sigma-Aldrich, USA) was reconstituted in ultrapure water and kept in stock concentration of 10 mM at −20 °C.

### 4.2 Cannabinoid Extraction

THC and CBD were extracted from Alaska (Sativa) and Rafael (Sativa) cannabis strains, respectively. The strains were contributed by “Tikun Olam”, which is one of the largest organizations in Israel that supplies medicinal cannabis to patients. The Alaska (Sativa) strain contains 20% THC and 1% CBD. Rafael (Sativa) contains 20% CBD and 1% THC. One gram of flowers from each strain listed above was frozen in liquid nitrogen, crushed, and dissolved in ethanol while stirring for 24 h. Mixtures were filtered to dispose of nonorganic precipitates. Ethanol was then evaporated, and the resulting oils were decarboxylated at 130 °C. Isolation of THC and CBD was performed with flash column chromatography with a mixture of hexane:ethyl acetate, 80%:20%. Fast Blue BB salt was used to detect THC and CBD on TLC plates. The THC CBD mixture was then subjected to preparative HPLC for cannabinoids separation, using isocratic elution (80%:20% acetonitrile:water). The fractions were then examined by LCMS to verify separation and purity (Figure S5). The cannabinoids were dissolved in ethanol and stored at stock concentrations of 100 mM at −20 °C, ensuring a final ethanol concentration in cell cultures below 0.1%. The use of ethanol rather than DMSO as a solvent, as seen in other cannabinoid-related studies, is based on data from in vitro reactions between carboplatin and DMSO, which inhibited carboplatin’s ability to initiate cell death and its cytotoxicity.^36^

### 4.3 Proliferation Assay—Carboplatin Sensitivity Model

To establish and confirm the model of three EOC cell lines representing a gradient of sensitivities to carboplatin, TOV-21G and OV-90 cells were seeded into 96 well plates at a density of 5000 cells per well. SKOV-3 cells were seeded into 96 well plates at a lower density of 3000 cells per well. All cell lines were incubated for 24 h, then treated with carboplatin at concentrations ranging from 0.05 to 160 μM, with control wells containing only fresh medium and no carboplatin. After the addition of carboplatin, cells were further incubated for 72 h, and cell proliferation was assessed using the XTT colorimetric assay (Sigma-Aldrich) following the manufacturer’s protocol. Briefly, 50 μL of XTT reagent was added to each well to a final well volume of 150 μL and further incubated for 4 h. Spectrophotometric absorbance was measured using a microplate reader at a wavelength of 490 nm with a reference wavelength of 650 nm. The concentration of carboplatin required to inhibit cell proliferation by 50% (IC_50_) was calculated for each cell line.

### 4.4 Cannabinoid Cytotoxicity Assay

To assess the cytotoxicity of CBD, THC, and their mixture on ovarian cancer cells, TOV-21G, OV-90, and SKOV-3 cells were seeded into 96-well plates as described in Section 2.3. All cell lines were incubated for 24 h and then treated with THC, CBD, or a mixture of both at concentrations of 0.05–100 μM. The mixture ratio was 1:1 molar; for example, a stated concentration of 80 μM corresponded to a final concentration of 40 μM THC and 40 μM CBD. Control wells contained no cannabinoids. After addition of the cannabinoids, cells were further incubated for 72 h, and cell proliferation was assessed using the XTT colorimetric assay (Sigma-Aldrich) following the manufacturer’s protocol, as described in Section 2.3.

### 4.5 Sensitivity Proliferation Assay

To examine the modulatory effect of cannabinoids on carboplatin efficacy in EOC cells, each cell line was seeded in 96-well plates with fresh medium for 24 h. THC, CBD, and their mixture were diluted in growth medium to a constant suboptimal concentration of 50% the respective IC_50_ for each cell line. The diluted cannabinoids were then added to the plates in the presence of increasing concentrations of carboplatin. Cells were further incubated for 72 h, and cell viability was assessed using the XTT viability assay. The IC_50_ value of carboplatin in the presence of each suboptimal cannabinoid was calculated and compared to the IC_50_ value of carboplatin alone.

### 4.6 Combination Index Proliferation Assay

To analyze the effects of carboplatin and cannabinoid combinations on EOC cells, each cell line was seeded in 96-well plates with fresh medium for 24 h. Cells were then treated with carboplatin, cannabinoids, and carboplatin-cannabinoid combinations at fixed equal ratios of their respective IC_50_, as described methodically by Chou et al.^37^ (Figure 6). Then, cells were further incubated for 72 h, and cell viability was assessed using the XTT viability assay. A combination index (CI) was calculated to assess the nature of the interaction between carboplatin and the various cannabinoids using the median-effect principle and the combination index equation as described by Chou et al. (Chou, 2010; Chou & Talalay, 1984):

$$CI=\left[{\left(Dcomb\right)}_{1}/{\left(Dcomb\right)}_{1}]+[{\left(Dcomb\right)}_{2}/{\left(Dcomb\right)}_{2}\right]$$

**Figure 6:**
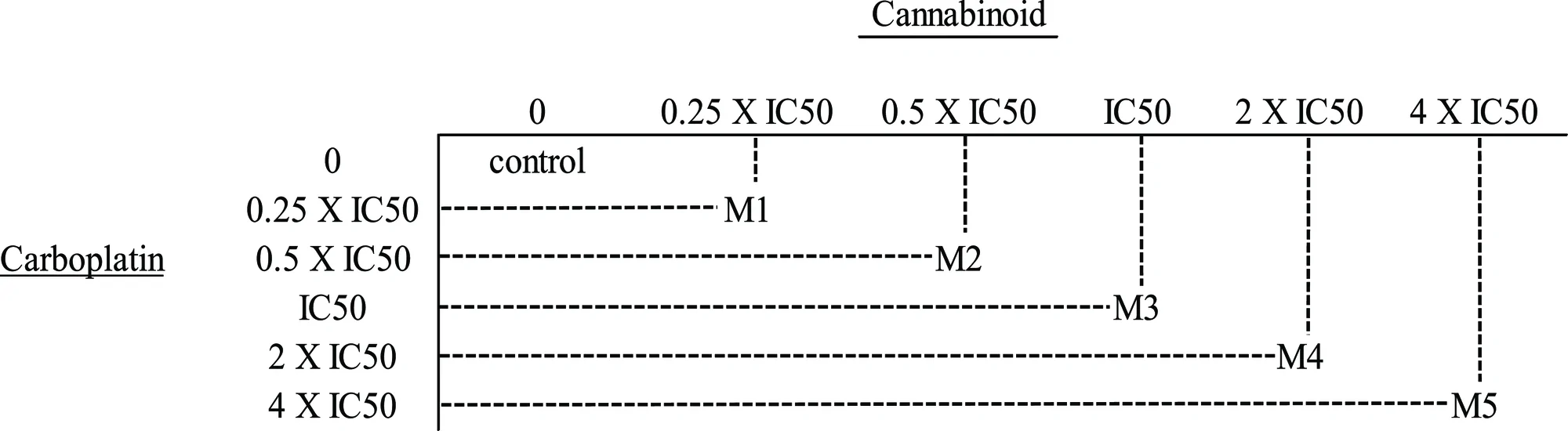
Combination index test outlay. A 16-point data test was performed for each cell line (5 wells of carboplatin, 5 wells of cannabinoids, 5 wells of mixed equal ratios, and 1 control well). M1-5 represent the 5 mixture wells with equal ratios of cannabinoids and carboplatin.

Where (*Dalone*)_1_ and (*Dalone*)_2_ represent concentrations of the drugs used individually to produce the IC_50_ value, and (*Dcomb*)_1_ and (*Dcomb*)_2_ represent concentrations of the drugs used in combination to produce the same effect. CI = 1 indicates an additive effect, CI < 1 a synergistic effect, and CI > 1 an antagonistic effect ct.^34,38^

### 4.7 Cannabinoid GPCRs Expression Analysis

RT-qPCR was performed using standard techniques. Briefly, for RNA isolation, roughly 1 × 10^6^ cells were washed twice with PBS, lysed with 0.5 mL GENEzol, and RNA was subsequently purified using the GENEzol TriRNA Pure Kit (Geneaid, Taiwan) according to the manufacturer’s protocol. cDNA was synthesized from 100 ng of RNA using Verso cDNA Synthesis Kit (Thermo Fisher Scientific, MA, USA). RT-qPCR was performed using a Rotor-gene 3000 instrument. The primers used in the reactions are listed in Table S1. Gene expression was also analyzed in silico by comparing expression data from normal human ovarian tissue in the GTEx database 34 (v8, transcript TPM, n = 180) and the CCLE database^39^ (updated January 2019). Data was extracted for the following genes: *CNR1*-ENSG00000118432.11; *CNR2*-ENSG00000188822.6; *GPR18*-ENSG00000125245.8; *GPR55*-ENSG00000135898.5; *GPR119*-ENSG00000147262.2.

### 4.8 DNA Damage Immunofluorescence Staining

To further assess the modulatory effect of THC and CBD on carboplatin efficacy, roughly 50,000 TOV-21G cells were seeded into μ-Slide 8 Well plates (ibidi) and incubated for 24 h. Ibidi slides were used to compensate for the very low cell adherence to poly-lysine-coated coverslips. Cannabinoids were then added at the same suboptimal concentration as described in Section 4.5, with or without carboplatin at its IC_50_ concentration. Cells were incubated for an additional 24 h, washed with PBS, and fixed with 4% paraformaldehyde. Slides were then stained with Phospho-Histone H2A.X Ab (Santa Cruz, sc-517348) and a Donkey antimouse Alexa Fluor 647 secondary antibody (abcam, ab150107) using standard techniques. Slides were filled with mounting medium containing DAPI and imaged on a Zeiss LSM880 confocal microscope. Images generated were analyzed using Fiji software. Carboplatin DNA damage on the cell line was compared in the absence and in the presence of the suboptimal cannabinoid concentrations.

### 4.9 Statistical Analysis

All experiments were performed in triplicate and values reported as mean ± SD. To calculate carboplatin and cannabinoid IC_50_ for each cell line, nonlinear regression dose–response curves were plotted using GraphPad Prism 7.0. The IC_50_ values were extracted from the regression curve. To assess the modulatory effect of cannabinoids on the efficacy of carboplatin on EOC cells, dose–response curves were calculated using GraphPad Prism 7.0, and the various IC_50_ values were compared using the extra sum of squares F test. The difference between groups was considered significant for *P* < 0.05. The CI values were calculated using the CompuSyn software for Quantitation of Synergism and Antagonism in Drug Combinations.

## Supplementary Material



## Notes

Primer sequences (Table S1), IC_50_ and CI values for effects of cannabinoids on cell lines (Tables S2 and S3), dose-response curves and median effect analysis for effects of cannabinoids on cell lines (Figures S1–S4), and LC/MS analysis of isolated and purified THC and CBD (Figure S5) (PDF)

## Abbrevations

THC: Δ9-tetrahydrocannabinol
CBD: cannabidiol
EOC: epithelial ovarian cancer
ECS: endocannabinoid system
GPCRs: G-protein-coupled receptors
CAM: complementary alternative medicine
TOV-21G: ovarian adenocarcinoma
OV-90: ovarian papillary serous adenocarcinoma
SKOV-3: ovarian adenocarcinoma
ATCC: American Type Culture Collection
CI: combination index
