# Supplementary material for: Evaluation of Phytocannabinoids as Antitumor Agents against Ovarian Cancer Cells in Combination with Carboplatin
Source: ACS Omega. 2026 Aug 26;11(35):52896–904. doi: 10.1021/acsomega.6c04745 (PMC13563602; doi:10.1021/acsomega.6c04745)
Supplement: Supplementary file 1 [file ao6c04745_si_001.pdf]

Supporting Information

Evaluation of Phytocannabinoids as Antitumor Agents against Ovarian Cancer Cells in Combination with Carboplatin

Uri Warter[1],[a] Yuval Yogev[1],[b] David C. Nwobodo,[c] Ksenia Lialin,[c] Danielle Mantin,[c] Mihai Meirovitz,[d] Michael M. Meijler*,[c] Amit Mayer*,[d]

[a] The Joyce & Irving Goldman Medical School, Faculty of Health Sciences, Ben-Gurion University of the Negev, Be'er Sheva, Israel
[b] The Morris Kahn Laboratory of Human Genetics at the National Institute of Biotechnology in the Negev and Faculty of Health Sciences, Ben-Gurion University, Be'er Sheva, Israel
[c] Department of Chemistry and the National Institute for Biotechnology in the Negev, Ben-Gurion University of the Negev, Be'er Sheva, Israel
[d] Division of Obstetrics and Gynecology, Gynecology Oncology Unit, Faculty of Health Sciences, Soroka University Medical Center, Ben-Gurion University of the Negev, Be'er Sheva, Israel

* Corresponding authors. meijler@bgu.ac.il; amitme@clalit.org.il

Table S1: Primer sequence for RT-qPCR of canabinoids GPRs expression analysis.

**Primers**	**Sequence**	
CB1	Forward	5-TCGTTCTAGCGGACAACCAG-3
	Reverse	5-AATCTCTTTGCCCCTTCGCA-3
CB2	Forward	5-TCCTGGGAGAGGACAGAAAACA-3
	Reverse	5-TGTCTAGAAGGCTTTGGGTTGTG-3
GPR18	Forward	5-TTGATGGACAGGAGTTTCTAAGT-3
	Reverse	5-TGAGCTGTTAAAAGGGACAGGT-3
GPR55	Forward	5-ACCACCAACAGTTGTGATTCAA-3
	Reverse	5- GATCAGACGGGGCTCCTTTC-3
GPR119	Forward	5- CCACTCGGAATCCCCATGTT-3
	Reverse	5-GAGGGTCAGCACGAAGTGAG-3

Table S2: IC_{50} values of carboplatin on TOV-21G, OV-90, and SKOV-3 cell lines

**Cell line**	**IC_{50} (µM)**
TOV-21G	10.49 ± 1.25
OV-90	18.30 ± 1.68
SKOV-3	35.51 ± 2.37

*Values represent the mean ± SD of at least 3 different experiments

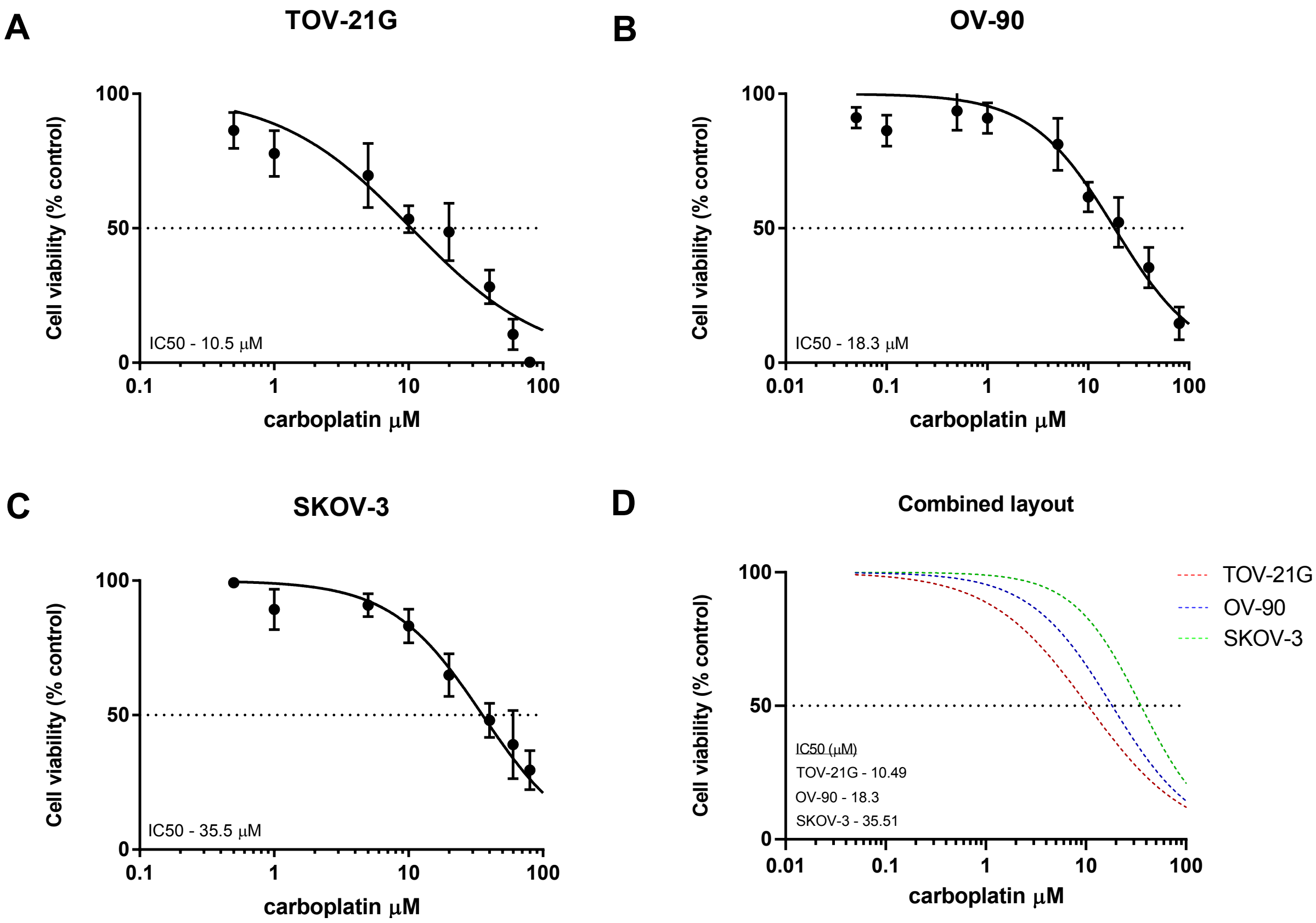

Fig. S1. Dose response curves of carboplatin on EOC cells. A, B, and C represent the IC_{50} values of carboplatin on TOV-21G, OV-90, and SKOV-3 cell lines, respectively. D represents an overlay comparison analysis of the effect of carboplatin on the three cell lines. Each data point represents the mean of at least three separate experiments, and IC_{50} values were significantly different between all 3 cell lines (PV<0.0001).

Table S3: CI values of cannabinoids and carboplatin at a fixed drug ration

CI at IC_{50}	CBD	THC	Mix
TOV-21G	1.82	1.89	2.06
OV-90	1.34	1.85	1.99
SKOV-3	1.90	1.71	1.27

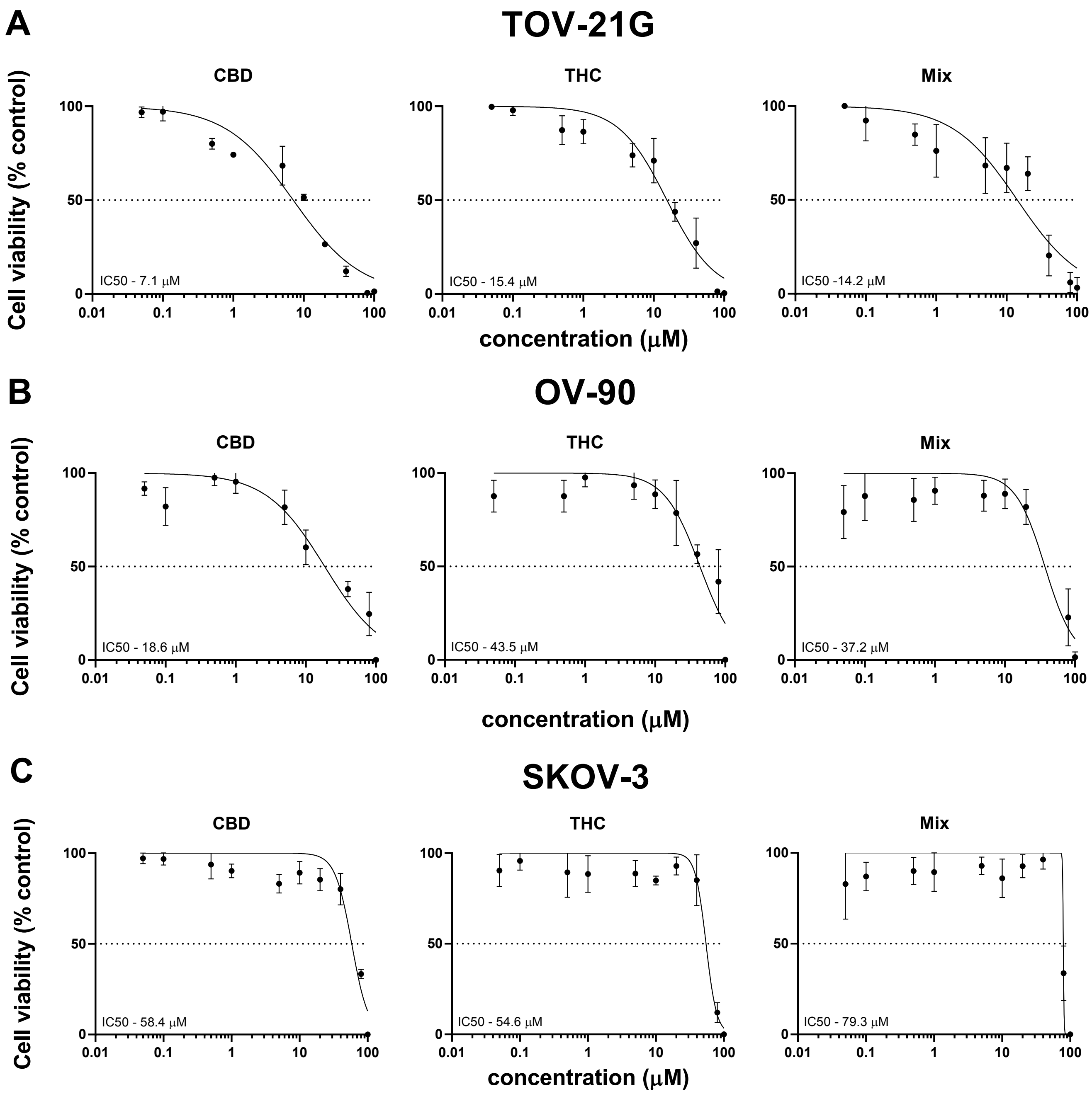

Fig. S2. Evaluation of cannabinoid cytotoxicity on EOC cells. A, B, and C represent the IC_{50} values of each cannabinoid and their mixture on TOV-21G, OV-90, and SKOV-3 cell lines, respectively.

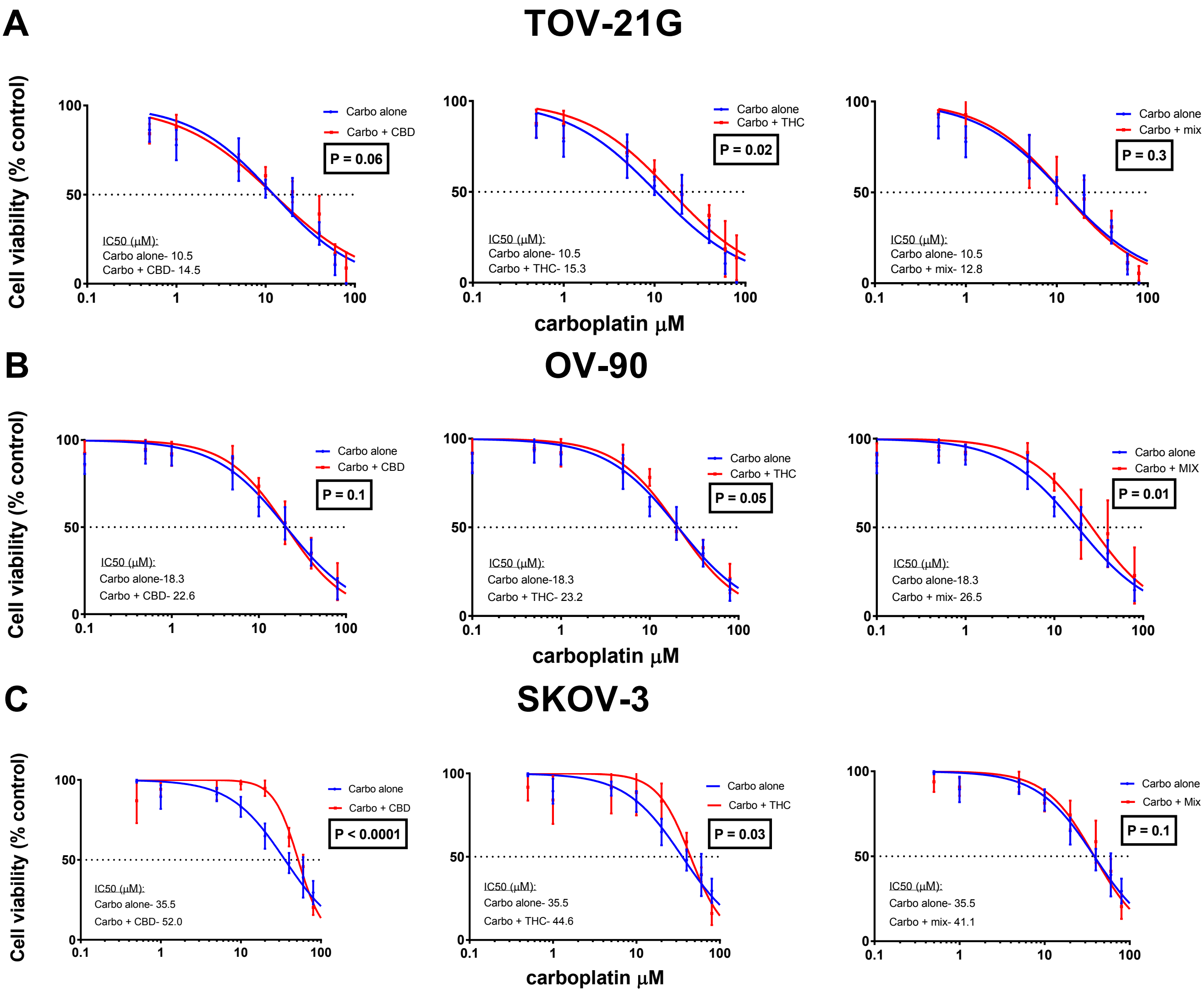

Fig S3. Comparison of the IC_{50} value of carboplatin in the presence of each suboptimal cannabinoid vs carboplatin alone for TOV-21G (A), OV-90 (B), and SKOV-3 (C) cell lines.

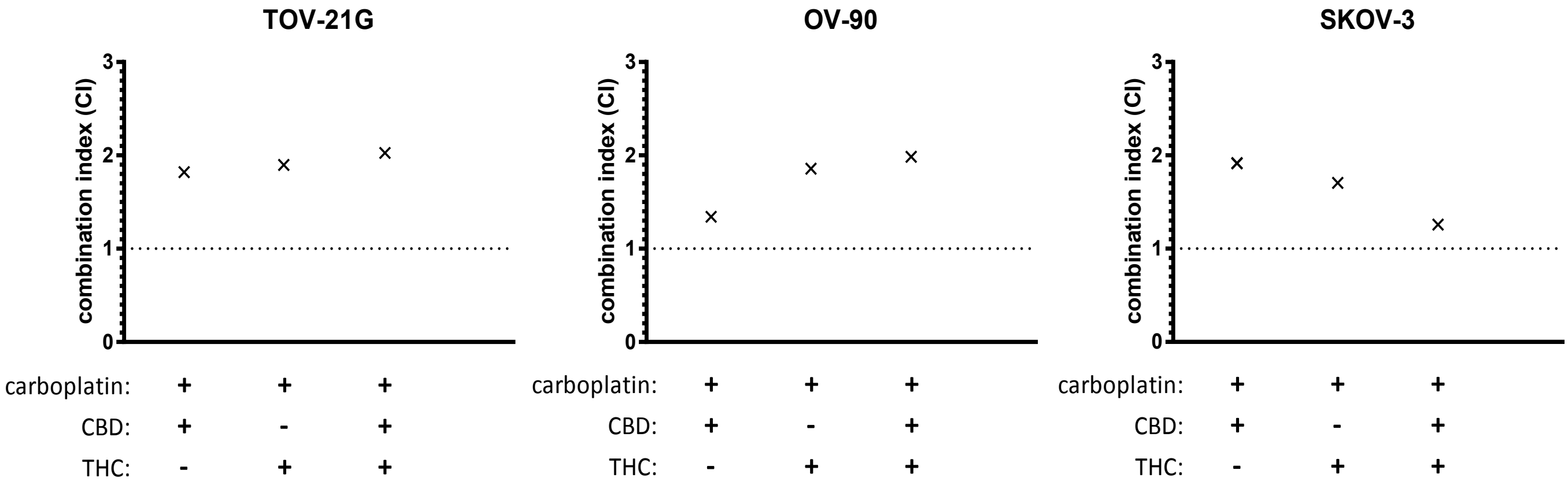

Fig S4. Median effect analysis plots of the cell lines. Synergistic interactions are characterized by points under the dotted line, whereas antagonistic interactions are characterized by points above the dotted line. Each data point represents the mean of at least three separate experiments

Isolation of cannabidiol (CBD) and tetrahydrocannabinol (THC) from cannabis flowers

The isolation of these specific cannabinoids was performed by decarboxylation of the oily extract of the different strains. After separation by flash column chromatography, using an 80:20 hexane: EtOAC mixture, two main compounds (THC and CDB) were isolated.

Fast blue BB salt is a diazonium salt that creates a complex with the cannabinoids and stains were developed using a spray on the TLC plate.

The THC and CBD spots were in close proximity on the TLC plate, and in order to separate them, HPLC was used. The gradient was isocratic, fractions were then examined by LC/MS to verify the separation. Fig. S5 shows the chromatograms and MS spectra belonging to the main peaks (THC: 8.6 min, CBD: 4.1 min). The peak at 1.68 min belongs to the solvent front.

Fig. S5. LC/MS analysis of purified THC and CBD

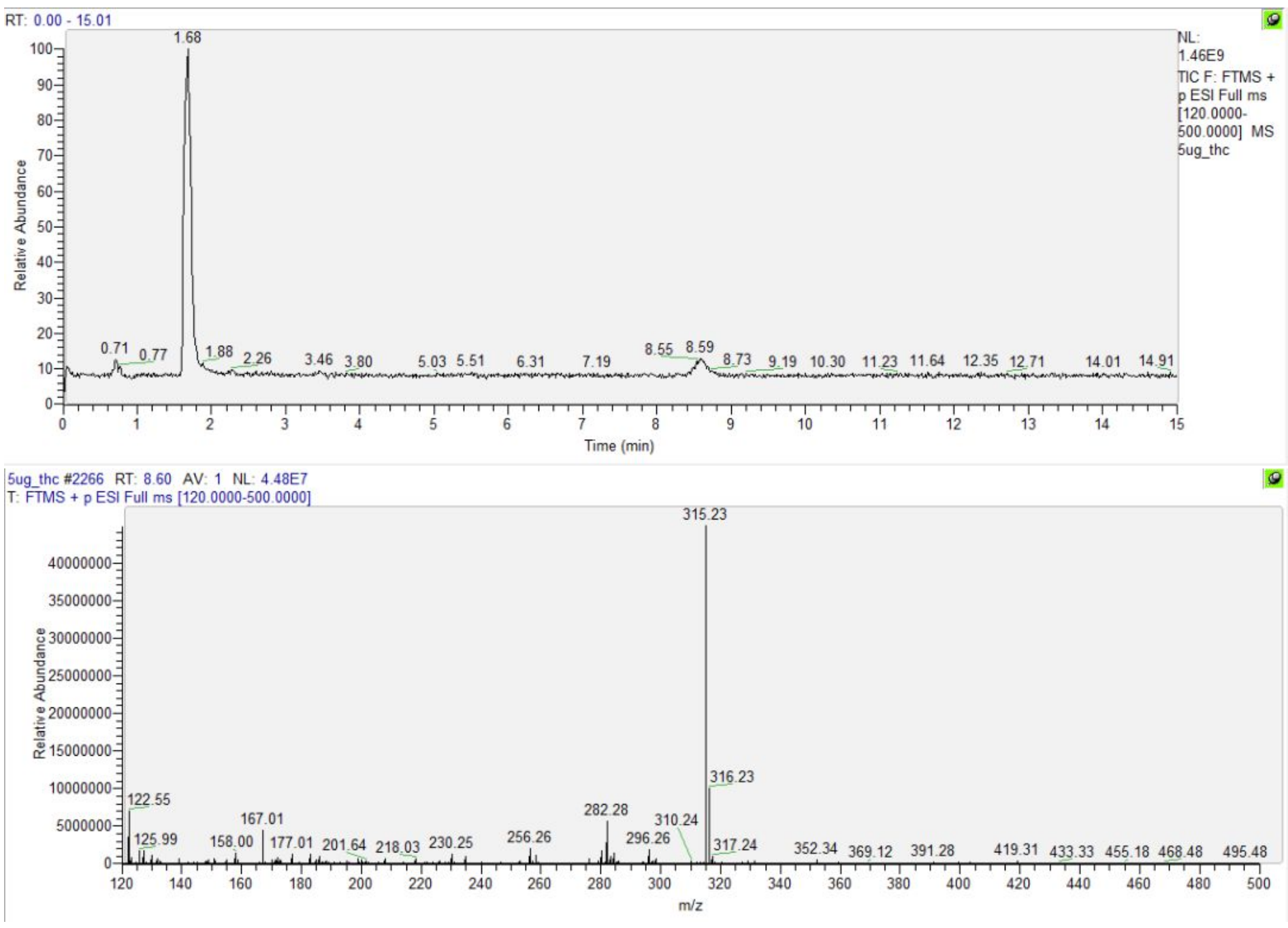

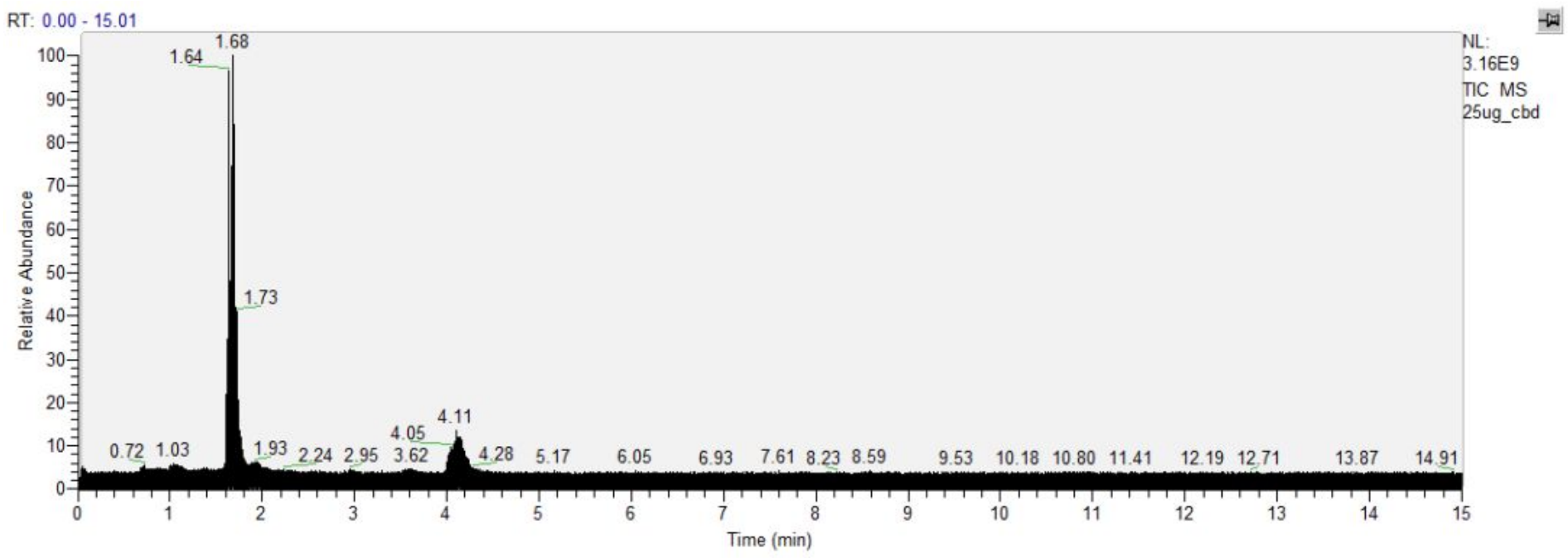

RT: 0.00 - 15.01
NL: 3.16E9 TIC MS 25ug_cbd
Relative Abundance
Time (min)
1.68
1.64
1.73
4.11
4.05
0.72
1.03
1.93
2.24
2.95
3.62
4.28
5.17
6.05
6.93
7.61
8.23
8.59
9.53
10.18
10.80
11.41
12.19
12.71
13.87
14.91

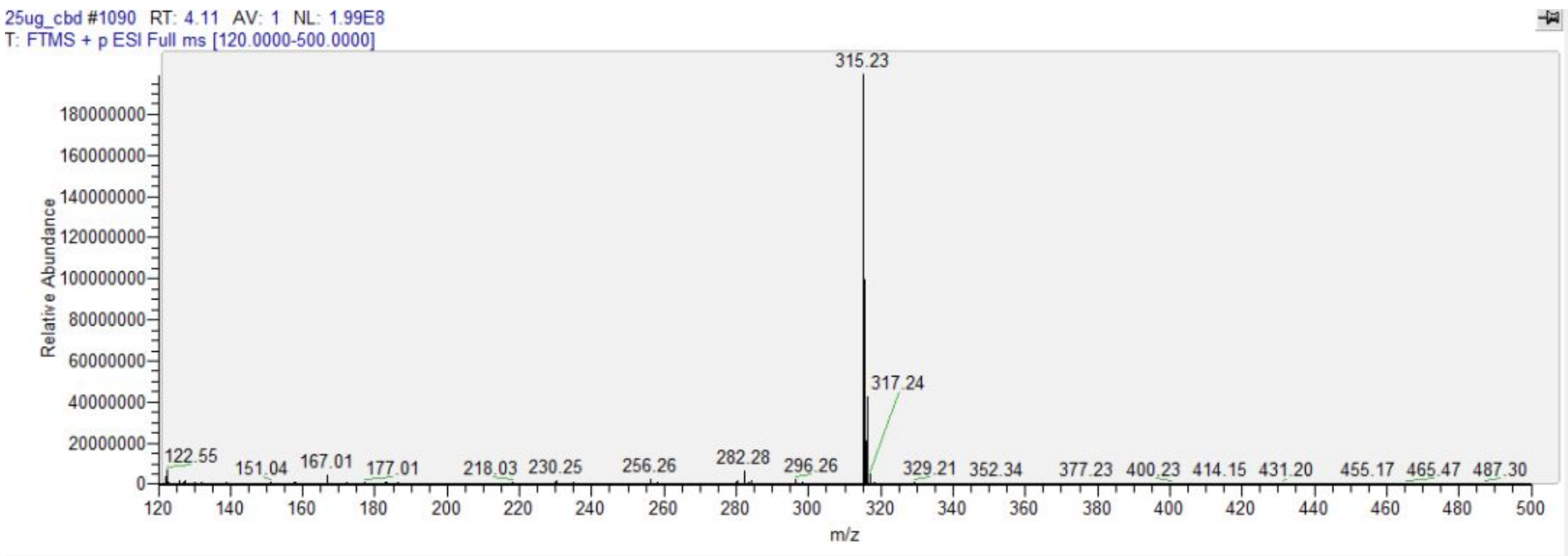

25ug_cbd #1090 RT: 4.11 AV: 1 NL: 1.99E8
T: FTMS + p ESI Full ms [120.0000-500.0000]
Relative Abundance
m/z
315.23
317.24
122.55
151.04
167.01
177.01
218.03
230.25
256.26
282.28
296.26
329.21
352.34
377.23
400.23
414.15
431.20
455.17
465.47
487.30
